# Predictors of in-hospital mortality following major lower extremity amputations in type 2 diabetic patients using artificial neural networks

**DOI:** 10.1186/s12874-016-0265-5

**Published:** 2016-11-22

**Authors:** Ana Lopez-de-Andres, Valentin Hernandez-Barrera, Roberto Lopez, Pablo Martin-Junco, Isabel Jimenez-Trujillo, Alejandro Alvaro-Meca, Miguel Angel Salinero-Fort, Rodrigo Jimenez-Garcia

**Affiliations:** 1 Preventive Medicine and Public Health Teaching and Research Unit, Health Sciences Faculty, Rey Juan Carlos University, Avda. de Atenas s/n, 28922 Alcorcón, Comunidad de Madrid Spain; 2 Research & Development Department, Artelnics, Spain; 3 Dirección Técnica de Docencia e Investigación, Gerencia Atención Primaria, Madrid, Comunidad de Madrid Spain

**Keywords:** Major lower extremity amputation, Type 2 diabetes, Charlson Comorbidity Index, Elixhauser Comorbidity Index, In-hospital mortality, Artificial neural networks

## Abstract

**Background:**

Outcome prediction is important in the clinical decision-making process. Artificial neural networks (ANN) have been used to predict the risk of post-operative events, including survival, and are increasingly being used in complex medical decision making. We aimed to use ANN analysis to estimate predictive factors of in-hospital mortality (IHM) in patients with type 2 diabetes (T2DM) after major lower extremity amputation (LEA) in Spain.

**Methods:**

We design a retrospective, observational study using ANN models. We used the Spanish National Hospital Discharge Database to select all hospital admissions of major LEA procedure in T2DM patients. Main outcome measures: Predictors of IHM using 4 ANN models: i) with all discharge diagnosis included in the database; ii) with all discharge diagnosis included in the database, excluding infectious diseases; iii) comorbidities included in the Charlson Comorbidities Index; iv) comorbidities included in the Elixhauser Comorbidity Index.

**Results:**

From 2003 to 2013, 40,857 major LEAs in patients with T2DM were identified with a 10.0% IHM. We found that Elixhauser Comorbidity Index model performed better in terms of sensitivity, specificity and precision than Charlson Comorbidity Index model (0.7634 vs 0.7444; 0.9602 vs 0.9121; 0.9511 vs 0.888, respectively). The area under the ROC curve for Elixhauser comorbidity model was 91.7% (95% CI 90.3–93.0) and for Charlson comorbidity model was 88.9% (95% CI; 87.590.2) *p* = 0.043. Models including all discharge diagnosis with and without infectious diseases showed worse results. In the Elixhauser Comorbidity Index model the most sensitive parameter was age (variable sensitive ratio [VSR] 1.451) followed by female sex (VSR 1.433), congestive heart failure (VSR 1.341), renal failure (VSR 1.274) and chronic pulmonary disease (VSR 1.266).

**Conclusions:**

Elixhauser Comorbidity Index is a superior comorbidity risk-adjustment model for major LEA survival prediction in patients with T2DM than Charlson Comorbidity Index model using ANN models. Female sex, congestive heart failure, and renal failure are strong predictors of mortality in these patients.

## Background

In the previous decade, incidence rates of diabetes-associated amputations have significantly decreased [1–5]. However, a major lower extremity amputation (LEA) is associated with a reduction of long-term survival and quality of life [6, 7].

In Spain, more than 5,000 major LEAs are performed yearly, 57% in patients with type 2 diabetes (T2DM) [8]. Despite improvements in long-term outcome, in-hospital mortality (IHM) following major LEA in patients with T2DM has remained stable from 2001 to 2012 with figures of 9.7% and 9.5% respectively [8, 9].

Outcome prediction is important in the clinical decision-making process. Older age, renal failure and congestive heart failure have been identified as independent factors for operative mortality following major LEA in Veterans Administration patients [10]. Wise et al. (2016) reported that preoperative septic shock and thrombocytopenia are independent risk factors for 30-day mortality after major LEA [11]. Other authors have described that age and vascular disease in patients with T2DM are important predictors that contribute to increase the mortality after major LEA [12]. However, to our knowledge, there are no studies investigating the predictive factors of in-hospital operative mortality in T2DM patients following major LEA.

Artificial neural networks (ANN) have been used to predict the risk of post-operative events, including survival, exploring complex relationships between preoperative variables survival in different surgical settings and are increasingly being used in complex medical decision making [13–16].

The aim of our study was to use ANNs analysis to estimate predictive factors of IHM in patients with T2DM after major LEA in Spain. We assessed the performance indices for ANN models in four scenarios: i) with all discharge diagnosis included in the database; ii) with all discharge diagnosis included in the database, excluding infectious diseases; iii) comorbidities included in the Charlson Comorbidities Index (CCI) and iv) comorbidities included in the Elixhauser Comorbidity Index (ECI).

## Methods

We have been used the Spanish national hospital database, from 2003 to 2013 to identify predictive factors of IHM in type 2 diabetic patients after major LEA. The database contained variables like sex, date of birth, admission date, discharge diagnoses (up to 14), procedures performed during the hospital stay (up to 20), length of hospital stay (LOHS) and IHM [17]. Diagnosis and procedures are codified according to the International Classification of Diseases, Ninth Revision, Clinical Modification (ICD-9-CM). Details on this database have been published previously [18, 19].

The study included all procedures of major amputation, defined as any LEA through or proximal to the ankle joint (ICD-9-CM codes 84.13–84.17) in discharges with a T2DM diagnosis (ICD-9-CM codes 250.x0; 250.*x*2) in any diagnosis position. We excluded all traumatic major LEAs (ICD-9-CM codes 895–897; 905.9; 928–929; 959 in any diagnosis field) and patients with type 1 diabetes (T1DM) (ICD-9-CM codes 250.x1; 250.x3).

The study sample included 40,857 patients who undergone major amputation procedure between January 1, 2003 and December 31, 2013.

### Development of the ANN models

To provide a good quality predictive model, the database has been randomly splitted in three independent subsets: 60% of the entire database for training the neural network (*n* = 24,513 cases), 20% for selecting those models with better generalization capabilities (*n* = 8,172 cases) and 20% for testing the model (*n* = 8,172 cases). The independent variables were age, sex, comorbidity and LOHS, and the dependent variable was outcome (IHM).

The ANN used in this study was a standard feed-forward, back-propagation neural network in which each input layer received information from the data, then it passed through the hidden layers and, finally, it arrived to the output layer. We used the quasi-Newton method in order to carry out the learning process (training algorithm) [20] and we applied model selection to find the optimal number of neurons in the hidden layer [21].

We elaborated four models as follow: model 1) including all discharge diagnosis in the database; model 2) all discharge diagnosis included in the database, except infectious diseases (ICD-9-CM codes 001 to 139); model 3) comorbidities included in the CCI [22] and finally; model 4) comorbidities included in the ECI [22].

### Statistical analyses

The unit of analysis was the individual patient with T2DM undergoing major LEA. A descriptive analysis was performed for all variables. Categories variables are shown as total numbers or proportions. Significant predictors were identified by bivariate analysis using chi square test, Student's *t*-test or Mann–Whitney *U* test when appropriate (*p* < 0.05, 2-tailed).

The predictive capabilities of the four models was analyzed using the area under the ROC curve (AUC) and the performance indices for ANN models described in Table 3 [23]. In this context, discrimination model is which to ability to distinguish patients who died from those who survived. Finally, once the model is trained, it can be studied the importance of the input variables by performing a sensitivity analysis. The sensitivity of an input variable is calculated dividing the sum squared error of the neural network responses without using the input variable by the sum squared error of the neural network responses using all the inputs. In this way, if the error without the input variable is lower than the error using all the inputs, the value of the sensitivity will be lower than 1 and if it is greater, the sensitivity will be greater than 1, as described previously by Shi et al. [24].

To estimate the 95% confidence interval and to compare AUC we used EPIDAT 4.2 software applying the methods described by DeLong et al. [25, 26].

The design of the four ANNs was performed using the predictive analytics solution Neural Designer, developed by the authors of this work. Statistical analyses were performed using the Stata MP version 10.1 statistical package (StataCorp LP, College Station, TX).

### Sensitivity analysis

In order to assess the possible misclassification of T1DM patients as T2DM or vice versa in the database the predictive capabilities of the CCI and the ECI were analyzed estimating the AUC including all patients with any diabetes ICD-9-CM codes (250.xx), beside Type 1 o Type 2.

## Results

Between January 1, 2003 and December 31, 2013, a total of 40,857 non-traumatic major LEAs (26,222 in men and 14,635 in women) corresponding to T2DM were identified in Spain. Almost 82% of the patients have ≥65 years of age (mean age 73.77 years, SD 10.26 years).

We found that 10.0% (*n* = 4,078) of the patients who died during the admission in hospital. The mean age of patients who died during their stay was significantly higher (76.58 years) than those survived (73.47 years).

The mean (SD) LOHS was similar, 23.10 (22.17) days in patients who died during their hospitalization and 23.78 (22.09) days in those who survive after the procedure (*P* > 0.05).

Table 1 shows the demographic characteristics and prevalence of comorbidities included in the CCI according to IHM after a major LEA in patients with T2DM.

**Table 1:** Demographic characteristics and prevalence of comorbidities included in the Charlson Comorbidity Index according to IHM due to major LEA in patients with T2DM in Spain, 2003-2013

	Prevalence		Condition IHM%		No condition IHM%		*p*-value
Men	26222	64.2	2363	9.0			<0.001
Women	14635	35.8	1715	11.7			
<50 years	805	2.0	33	4.1			<0.001
50–64 years	6702	16.4	439	6.6			
65–79 years	20334	49.8	1862	9.2			
≥80 years	13016	31.9	1744	13.4			
Myocardial infarction	2472	6.05	393	15.9	3685	9.6	<0.001
Congestive heart failure	4384	10.73	1020	23.3	3058	8.4	<0.001
Peripheral vascular disease	28120	68.83	2648	9.4	1430	11.2	<0.001
Cerebrovascular disease	3993	9.77	524	13.1	3554	9.6	<0.001
Dementia	2373	5.81	329	13.9	3749	9.7	<0.001
Chronic pulmonary disease	4706	11.52	570	12.1	3508	9.7	<0.001
Rheumatoid disease	523	1.28	51	9.8	4027	10.0	0.860
Peptic ulcer disease	375	0.92	56	14.9	4022	9.9	<0.001
Mild liver disease	1114	2.73	149	13.4	3929	9.9	<0.001
Hemiplegia or paraplegia	248	0.61	22	8.9	4056	10.0	0.559
Renal disease	7147	17.49	1054	14.7	3024	9.0	<0.001
Any malignancy	854	2.09	95	11.1	3983	10.0	0.260
Moderate or severe liver disease	202	0.49	48	23.8	4030	9.9	<0.001
Metastatic solid tumor	157	0.38	31	19.7	4047	9.9	<0.001
AIDS/HIV	27	0.07	2	7.4	4076	10.0	0.655

*P* value comparing IHM between those with and without the condition. Any malignancy, including lymphoma and leukemia, except malignant neoplasm of skin

We found that 68.83% of the patients had peripheral vascular disease, 17.49% have renal disease and 11.52% have chronic pulmonary disease these were the three more common chronic conditions.

In our study IHM in women with T2DM was more common than in diabetic men (11.7% vs. 9.0%, *p* < 0.05). IHM was significantly higher in older patients (13.4% in ≥80 years vs. 4.1% in <50 years). Patients with myocardial infarction, congestive heart failure, peripheral vascular disease, cerebrovascular disease, dementia, chronic pulmonary disease, peptic ulcer disease, mild liver disease, renal disease, moderate or severe liver disease or metastatic solid tumour had significantly higher IHM than those without these conditions (Table 1). Over 20% of the patients suffering moderate or severe liver disease (23.8%) and congestive heart failure (23.3%) died in the hospital after a major LEA.

As can be seen in Table 2 when we analyse comorbidity included in the ECI we find that peripheral vascular disease and hypertension (47% complicated and 1.8% uncomplicated) were the more prevalent diseases in diabetic patients who underwent LEAs. All patients with any ECI (except those with paralysis, hypothyroidism, peptic ulcer disease excluding bleeding, AIDS/HIV, rheumatoid arthritis/collagen vascular diseases, obesity, blood loss anemia, alcohol abuse or drug abuse) have significantly higher IHM than those without these conditions (Table 2). Beside congestive heart failure (23.3%) patients suffering fluid and electrolyte disorders (29.9%), coagulopathy (24.8%) and pulmonary circulation disorders (21.6%) showed the highest IHM.

**Table 2:** Prevalence of comorbidities included in the Elixhauser Comorbidity Index according to IHM due to major LEA in patients with T2DM in Spain, 2003-2013

Comorbidities	Prevalence		Condition IHM		No condition IHM		*p*-value
Congestive heart failure	4384	10.7	1020	23.3	3058	8.4	<0.001
Cardiac arrhythmias	7552	18.5	1079	14.3	2999	9.0	<0.001
Valvular disease	1716	4.2	244	14.2	3834	9.8	<0.001
Pulmonary circulation disorders	500	1.2	108	21.6	3970	9.8	<0.001
Peripheral vascular disorders	28120	68.8	2648	9.4	1430	11.2	<0.001
Hypertension, uncomplicated	19204	47.0	1616	8.4	2462	11.4	<0.001
Hypertension, Complicated	5220	12.8	720	13.8	3358	9.4	<0.001
Paralysis	248	0.6	22	8.9	4056	10.0	0.559
Other neurological disorders	1268	3.1	174	13.7	3904	9.9	<0.001
Chronic pulmonary disease	4706	11.5	570	12.1	3508	9.7	<0.001
Hypothyroidism	867	2.1	84	9.7	3994	10.0	0.771
Renal failure	7125	17.4	1052	14.8	3026	9.0	<0.001
Liver disease	1316	3.2	197	15.0	3881	9.8	<0.001
Peptic ulcer disease excluding bleeding	223	0.5	23	10.3	4055	10.0	0.868
AIDS/HIV	27	0.1	2	7.4	4076	10.0	0.655
Lymphoma	70	0.2	2	2.9	4076	10.0	0.047
Metastatic cancer	157	0.4	31	19.7	4047	9.9	<0.001
Solid tumor without metastasis	817	2.0	107	13.1	3971	9.9	0.003
Rheumatoid arthritis/collagen vascular diseases	686	1.7	72	10.5	4006	10.0	0.650
Coagulopathy	270	0.7	67	24.8	4011	9.9	<0.001
Obesity	1898	4.6	195	10.3	3883	10.0	0.663
Weight Loss	542	1.3	106	19.6	3972	9.9	<0.001
Fluid and electrolyte disorders	1043	2.6	312	29.9	3766	9.5	<0.001
Blood loss anemia	209	0.5	30	14.4	4048	10.0	0.034
Deficiency anemia	922	2.3	112	12.1	3966	9.9	0.026
Alcohol abuse	1841	4.5	165	9.0	3913	10.0	0.136
Drug abuse	57	0.1	5	8.8	4073	10.0	0.761
Psychoses	292	0.7	25	8.6	4053	10.0	0.417
Depression	1429	3.5	112	7.8	3966	10.1	0.006

*P* value comparing IHM between those with and without the condition

The ANN-based approaches provided the 3-layer networks and the relative weights of neurons used for predicting IHM. Table 3 shows the ANN models in terms of binary classification tests. The data analysis showed that ANN model 3 and ANN model 4 have the best accuracy rate (0.8612 and 0.8307) and ECI model performed better in terms of sensitivity, specificity and precision than CCI model (0.7634 vs 0.7444; 0.9602 vs 0.9121; 0.9511 vs 0.888, respectively).

**Table 3:** Comparison of performance indices for Artificial Neural Network models when using new data sets for predicting in hospital mortality rate in patients with T2DM after major LEA in Spain

Indices	Description	Model 1	Model 2	Model 3	Model 4
Accuracy rate	Ratio of instances correctly classified	0.807	0.794	0.830	0.861
Error rate	Ratio of instances misclassified	0.192	0.205	0.169	0.138
Sensitivity	Portion of actual positive which are predicted positive	0.775	0.801	0.744	0.763
Specificity	Portion of actual negative predicted negative	0.838	0.786	0.912	0.960
Precision	Portion of predicted positive which are actual positive	0.821	0.787	0.888	0.951
Positive likelihood	Likelihood that a predicted positive is an actual positive	4.794	3.756	8.476	19.23
Negative likelihood	Likelihood that a predicted negative is an actual negative	3.733	3.962	3.569	4.06
F1 score	Harmonic mean of precision and sensitivity	0.797	0.794	0.810	0.847
False positive rate	Portion of actual negative which are predicted positive	0.161	0.213	0.087	0.039
False discovery rate	Portion of predicted positive which are actual negative	0.178	0.212	0.111	0.048
False negative rate	Portion of actual positive which are predicted negative	0.224	0.198	0.255	0.236
Negative predictive value	Portion of predicted negative which are actual negative	0.795	0.800	0.790	0.800
Matthews correlation	Correlation between the targets and the outputs. It takes a value between -1 and +1	0.615	0.588	0.668	0.737
Informedness	Probability that the prediction method will make a correct decision as opposed to guessing	0.613	0.588	0.656	0.723
Markedness	Probability of predicting the classifier labels from the real classes.	0.659	0.574	0.801	0.911

Model 1: all discharge diagnosis included in the database. Model 2: all discharge diagnosis included in the database, except infectious diseases (ICD-9-CM codes 001 to 139). Model 3: comorbidities included in the Charlson Comorbidities Index. Model 4: comorbidities included in the Elixhauser Comorbidities Index

**Fig. 1:**
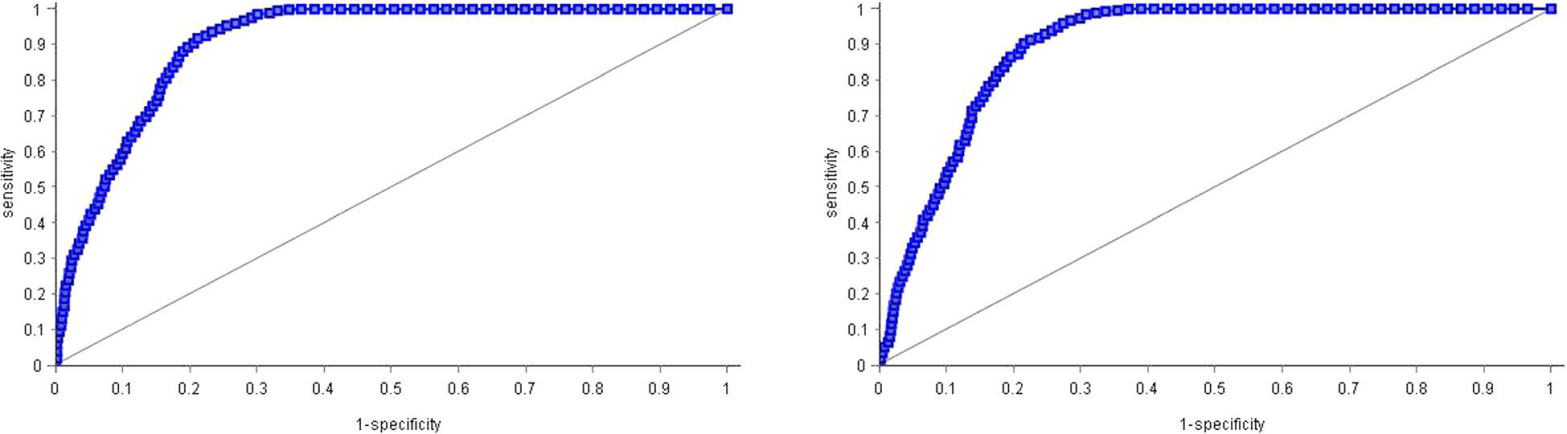
Area under the ROC curve of the Charlson comorbidities index model and the Elixhauser comorbidities index model in predicting the in-hospital mortality rate after major LEA in patients with T2DM in Spain

Compared with CCI model, ECI model showed a significantly better (*p* = 0.043) area under the ROC curve (91.7% [95% CI 90.3–93.0] vs 88.9% [95% CI; 87.5–90.2]) as can been seen in Fig. 1. The area under the curve for models 1 and 2 were 0.87 and 0.867, respectively, showing that these models are less accurate to predict IHM after major LEAS using ANN.

The selection data set was also used to calculate the variable sensitive ratio (VSR). Table 4 presents the VSR values for the outcome variable (IHM) regarding CCI and ECI comorbidities. In the ECI network for predicting IHM, the most sensitive parameter was age (VSR 1.451) followed by female sex (VSR 1.433), congestive heart failure (VSR 1.341), renal failure (VSR 1.274) and chronic pulmonary disease (VSR 1.266). Age, female sex, myocardial infarction, renal disease and congestive heart failure were the predictors of IHM with the CCI network. All VSR exceeded 1, which indicated that the network performs better when all variables were considered.

**Table 4:** Global sensitivity analysis of the Charlson comorbidities index model and the Elixhauser comorbidities index model in predicting the in-hospital mortality rate after major LEA in patients with T2DM in Spain

	Charlson model		Elixhauser model	
Variable ranking	Variable	VSR	Variable	VSR
1st	Age	1.577	Age	1.451
2nd	Female	1.559	Female	1.433
3rd	Myocardial infarction	1.477	Congestive heart failure	1.341
4th	Renal disease	1.456	Renal failure	1.274
5th	Congestive heart failure	1.447	Chronic pulmonary disease	1.266
6th	Moderate or severe liver disease	1.412	Liver disease	1.262
7th	Metastatic solid tumor	1.362	Metastatic cancer	1.246

*VSR* Variable sensitivity ratio

The sensitivity analysis, including all diabetic patients beside if codified as Type 1(*n* = 1531; 3.61%) or Type 2 (*n* = 40,857; 96.39%), showed that the AUC for the ECI was 90.1% (95% CI 88.9–91.3) and for the CCI was 86.9% (95% CI 85.7%–88.2%) (*p* = 0.026).

## Discussion

To our knowledge, this study is the first to use a nationwide population-based discharge database to train and test an ANN for predicting factors of IHM in type 2 diabetic patients after major LEAs. We assessed and compared the two most commonly used comorbidity risk adjustment models in surgery, the Charlson and Elixhauser measures, regarding their ability to predict in-hospital death [22].

Like in other surgical procedures it is necessary to appropriately adjust for T2DM patient risk, recognizing that the underlying nature of some patients’ conditions may make them more likely than others to experience poor outcomes. Using ANNs we found that the ECI is a superior comorbidity risk-adjustment model for major lower extremity amputation in-hospital mortality compared with the CCI measure. Although differences in the area under the ROC curve between the two comorbidity-based measures were small, it has been noted that even slight improvements in area under the ROC curve for such indexes can translate into quantificable reductions in confounding bias [27]. Overall, the area under the ROC curve for inpatient mortality for the Charlson and Elixhauser comorbidity-based measures in our study were high and comparable to those described in other patient population [28, 29].

Major LEA in T2DM patients is associated with significant mortality. Our IHM of 10.0% is consistent with previously reported data ranging from 7% to 12.4% [7, 10, 30–33]. Investigation of underlying influences from health services, surgical decisions and patient motivations behind decisions to amputate might help to explain some of the differences in post-operative mortality rates between studies [34].

The effect of advancing age on IHM is also highlighted in this study, as has been well established by other authors [7, 10, 30]. Older age is associated with high prevalence of comorbid conditions, mainly cardiovascular and renal diseases, both of which are also associated with higher mortality rates [32, 35].

Despite having lower incidence rates of major LEAs several studies, including ours, suggest that women have higher mortality rates associated with diabetes-related LEAs [36, 37]. In US have reported that women had higher IHM associated with diabetes-related LEAS (37.7 IHM/1000 amputations vs. 29.7/1000 amputations [38]. Some previous research suggests higher LEA-associated cardiovascular disease mortality risk, including atherosclerotic complications, among women with LEAs [35, 38–40].

Several studies have evaluated specific comorbid diseases associated with reduced survival after major LEA [30–35, 38, 40]. Patients who needed major amputations suffered from a more severe cardiovascular disease than others [36]. Previous studies in Spain, significant risk factors for IHM were cardiac and respiratory complications and first episode of amputation [41]. Schofield et al. concluded that diabetic amputees have a greater risk of heart failure (OR: 2.26; 95% CI 1.12–4.57) further amputation and death than nondiabetic amputees [12] and highlighted the need for a more aggressive approach in the management of cardiovascular risk factors in those who undergo amputation and have diabetes. A recent study in the Canadian population reported that predictors of prolonged hospital stay included cardiovascular risk factors, such as diabetes, hypertension, ischemic heart disease, congestive heart failure or hyperlipidemia [42].

Different studies have identified renal disease as a risk factor for a significantly lower long-term survival after a major LEA [6, 36, 43–46]. Recently, Assi et al. reported that patients with chronic kidney disease have higher risk (OR 2.27; 95% CI 1.02–5.06) of postoperative long-term mortality and concluded that how CKD contributes to worse survival is unclear [31].

Other factor that has been found associated to mortality after amputations among people with diabetes was chronic pulmonary disease. Shah et al. demonstrated that several independent factors were detrimental to survival (at 24 months) including chronic obstructive pulmonary disease (hazard ratio [HR] 1.82, *P* = 0.002), dialysis dependence (HR 2.50, *P* < 0.001), high cardiac risk (HR 2.20, *P* < 0.001), and guillotine amputation (HR 2.49, *P* = 0.004) [33].

The strength of our study lies in its large sample size and in the use of ANN models. Different studies indicated that ANN models can simultaneously process numerous variables and can consider outliers and nonlinear interactions among variables. Therefore, whereas conventional statistics reveal parameters that are significant only for the overall population, the ANN model includes parameters that are significant at the individual level even if they are not significant in the overall population [13–16].

One of the major limitations of our study is that our data source was the CMBD, which contains administrative discharge data for hospitalizations in Spain and uses information the physician included in the discharge report. In our data we have no information about detailed clinical variables like glycemic control or lifestyle variables like smoking habits. Nevertheless, the CMBD, are periodically audited and the validity of our dataset has been assessed and shown to be useful for health research [47].

Another possible limitation is that a misclassification bias may occur and some patients suffering T2DM may have been recorded as T1DM or vice versa. In our investigation we excluded T1DM patients because in our database only 1531 patients had the codes for T1DM recorded, representing the 3.61% of all mayor amputations analyzed. Furthermore, patients with T1DM were significantly younger (mean age 66.56 years vs. 73.77 years; *p* < 0.001) and had less co-morbidities (mean CCI 1.25 vs. 1.39 and mean ECI 1.90 vs. 2.27; both *p* < 0.001) than those with T2DM. However, the results of the sensitivity analysis showed very similar results suggesting that the misclassification bias would not affect the main conclusions of our study.

## Conclusions

In conclusion, using ANN models we found that ECI method is a superior comorbidity risk-adjustment model for major LEA survival prediction in patients with T2DM than Charlson comorbidity. The global sensitivity analysis with the ECI model showed that the more predictors of IHM after major LEA procedure were older age followed by female sex, congestive heart failure, renal failure, chronic pulmonary disease, liver disease and metastatic cancer. The predictors analyzed in this study could be addressed in preoperative and postoperative health care of patients with T2DM candidates for major LEA.

## Abbreviations

ANN: Artificial neural networks
CCI: Charlson Comorbidities Index
CMBD: Spanish National Hospital Discharge Database, *Conjunto Mínimo Básico de Datos*
ECI: Elixhauser comorbidity index
HR: Hazard ratio
ICD-9-CM: International classification of diseases, ninth revision, clinical modification
IHM: In-hospital mortality
LEA: Lower extremity amputation
LOHS: Length of hospital stay
SMHSSE: Spanish ministry of health, social services, and equality
T1DM: Type 1 diabetes mellitus
T2DM: Type 2 diabetes mellitus
VSR: Variable sensitive ratio
